# "Single nucleotide polymorphisms of the *OPG/RANKL *system genes in primary hyperparathyroidism and their relationship with bone mineral density"

**DOI:** 10.1186/1471-2350-12-168

**Published:** 2011-12-20

**Authors:** María Piedra, María T García-Unzueta, Ana Berja, Blanca Paule, Bernardo A Lavín, Carmen Valero, José A Riancho, José A Amado

**Affiliations:** 1 Endocrinology Service, University Hospital "Marqués de Valdecilla" University of Cantabria-IFIMAV. Avda. de Valdecilla, Santander 39008. SPAIN; 2 Clinical Biochemistry Service, University Hospital "Marqués de Valdecilla" University of Cantabria-IFIMAV. Avda. de Valdecilla, Santander 39008. SPAIN; 3 Internal Medicine Service, University Hospital "Marqués de Valdecilla". University of Cantabria-IFIMAV. Avda. de Valdecilla, Santander 39008. SPAIN

## Abstract

**Background:**

Primary hyperparathyroidism (PHPT) affects mainly cortical bone. It is thought that parathyroid hormone (PTH) indirectly regulates the activity of osteoclasts by means of the osteoprotegerin/ligand of the receptor activator of nuclear factor-κβ (OPG/RANKL) system. Several studies have confirmed that *OPG *(osteoprotegerin) and *RANKL *(ligand of the receptor activator of nuclear factor-κβ) loci are determinants of bone mineral density (BMD) in the general population. The aim of this study is to analyze the relationship between fractures and BMD and the rs3102735 (163 A/G), rs3134070 (245 T/G) and rs2073618 (1181 G/C) SNPs of the *OPG *and the rs2277438 SNP of the *RANKL*, in patients with sporadic PHPT.

**Methods:**

We enrolled 298 Caucasian patients with PHPT and 328 healthy volunteers in a cross-sectional study. We analyzed anthropometric data, history of fractures or renal lithiasis, biochemical determinants including markers for bone remodelling, BMD measurements in the lumbar spine, total hip, femoral neck and distal radius, and genotyping for the SNPs to be studied.

**Results:**

Regarding the age of diagnosis, BMI, menopause status, frequency of fractures or renal lithiasis, we found no differences between genotypes in any of the SNPs studied in the PHPT group. Significant lower BMD in the distal radius with similar PTH levels was found in the minor allele homozygotes (GG) compared to heterozygotes and major allele homozygotes in both *OPG *rs3102735 (163 A/G) and *OPG *rs3134070 (245 T/G) SNPs in those with PHPT compared to control subjects. We found no differences between genotypes of the *OPG *rs2073618 (1181 G/C) SNP with regard to BMD in the PHPT subjects. In the evaluation of rs2277438 SNP of the *RANKL *in PHPT patients, we found a non significant trend towards lower BMD in the 1/3 distal radius and at total hip in the minor allele homocygotes (GG) genotype group versus heterocygotes and major allele homocygotes (AA).

**Conclusions:**

Our study provides the first evaluation of the relationship between SNPs of the *OPG/RANK *system and sporadic PHPT. Subjects with PHPT and minor homocygote genotype (GG) for the *OPG *rs3102735 (163 A/G) and *OPG *rs3134070 (245 T/G) SNPs have lower BMD in the distal radius, and this association does not appear to be mediated by differences in PTH serum levels.

## Background

Primary hyperparathyroidism (PHPT) is a common endocrine disorder, usually sporadic and asymptomatic, characterized by inappropriate hypersecretion of parathyroid hormone (PTH) and hypercalcemia. PHPT expression in bone involves mainly cortical bone such as the distal radius, with preservation of cancellous bone [1-8] which suggests that the response to high levels of PTH may differ depending on the skeletal structure [9], having anabolic effects in cancellous bone and catabolic actions at cortical sites. These findings in the pattern of bone mineral density (BMD) in PHPT are the opposite of those found in postmenopausal osteoporosis (OP). However it has been demonstrated that a group of patients with PHPT have lumbar OP that can improve after parathyroidectomy [10].

It is thought that PTH indirectly stimulates osteoclasts through neighbouring osteoblasts by inducing the ligand of the receptor activator of nuclear factor-κβ (RANKL) expression [7,11]. RANK is expressed on the osteoclasts cell membrane and RANKL is a cytokine present on the membranes of osteoblasts and when binding with RANK, osteoclast differentiation, activation and survival are stimulated. RANK-RANKL interactions, and its effects, are prevented if RANKL binds with osteoprotegerin (OPG), a soluble receptor secreted by the osteoblasts. Genome wide association studies tested candidate genes for their association with bone mineral density (BMD) and found that the *OPG *gene is a determinant of fractures and BMD in both the spine and the hip [12,13] and that *RANKL *locus influences BMD in both the spine and the hip also [13].

A number of studies analyzing the association between different single nucleotide polymorphisms (SNPs) of the *OPG-RANK-RANKL *system genes and bone density have been carried out in the osteoporotic and general populations, including a recent meta-analysis [14], but none of these studied sporadic PHPT. SNPs rs3102735 (163 A/G) [15-17] and rs3134070 (245 T/G) [15,18] of the *OPG *have been studied, showing lower BMD or higher frequency of fractures related to the minor allele (G) at different bone sites, mainly in postmenopausal women. Regarding the rs2073618 (1181 G/C) SNP of the *OPG*, the minor allele (C) has been related to higher BMD [15,18-24]. Other studies could not demonstrate these associations [25-28]. Among the SNPs of the *RANKL *studied, the minor allele (G) of the rs2277438 has been related to a higher femoral neck compression strength index [29] but not to BMD [19,29,30]. The aim of this study is to analyze the relationship between BMD and fractures and the three aforementioned SNPs of the *OPG *and the rs2277438 SNP of the *RANKL*, in patients with sporadic primary hyperparathyroidism, a model of chronic PTH hyperstimulation of the skeleton.

## Methods

The study population consisted of 298 Caucasian patients with sporadic PHPT. Cases were defined by chronic hypercalcemia (total and/or ionic calcium) plus high levels of PTH or plus inappropriately normal PTH levels which means hypercalcemia and PTH values at the upper limit of the normal range. We excluded the few cases of PHPT that were part of a Multiple Endocrine Neoplasia Syndrome. After cautiously reviewing the family clinical history we also excluded cases of possible hereditary isolated PHPT.

In order to study the allelic distribution in PHPT *versus *healthy population, 328 volunteers were enrolled through face to face and written requests from hospital workers, and through civic associations, religious groups and geriatric residences. Written informed consent was obtained from participants. This study was approved by the Ethics Committee of our hospital (University Hospital "Marqués de Valdecilla", Santander, Spain). Subjects with history of diseases known to affect skeletal homeostasis, with non-Spanish ancestry, or who were taking drugs which interfere with bone metabolism (biphosphonates, strontium ranelate, corticosteroids, anti-epileptics, estrogens, thiazides, hormonal replacement therapy with estrogens in women beyond 55 years old) were excluded. Fracture condition was defined by any traumatic or spontaneous fracture at any location except the nose, toe, head, jaw, skull and hands. The renal lithiasis condition was defined by any detected lithiasis by radiological exploration with or without symptoms.

### Measurement of BMD

MD was quantified using X-ray absorptiometry (DXA, Hologic, Walthan, MA, USA) in the lumbar spine (L_2-4_), femoral neck, total hip and radius. The BMD measurement variation coefficient was 1.2% in lumbar spine, total hip locations and radius projection, and 1.4% in the femoral neck.

### DNA Analysis

Genomic DNA was extracted from venous blood (buffy coat) by the Qiagen method (Hilden, Germany) and stored at -40°C until analyzed. Polymerase chain reaction (PCR) products were amplified in a 5 μl reaction following the instructions of the manufacturer in an Applied Thermal Cycler 9700 (Applied Biosystems). Cycling conditions consisted of an initial denaturation step at 95°C for 10 minutes and 48 cycles of denaturation at 92°C for 30 seconds and annealing for 1 minute at 60°C. After amplification, end-point fluorescence reading and allele identification were carried out with an ABI 7300 sequence detector (Applied Biosystems). Random samples (8% of the total samples) were analysed twice for quality control.

**Figure 1:**
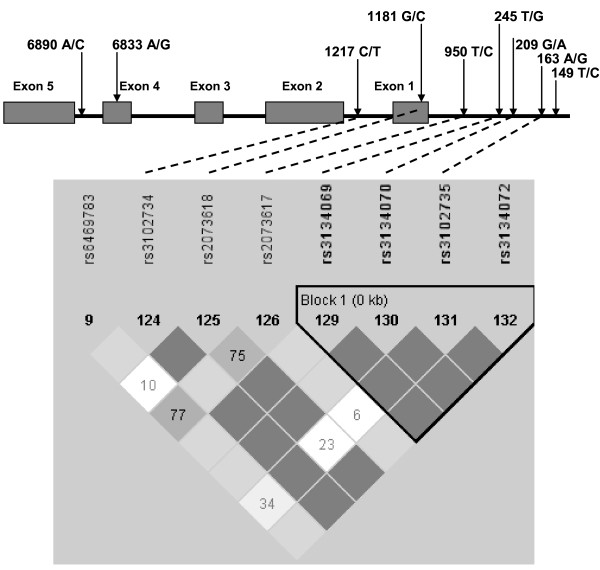
**Polymorphisms in the proximal region of the OPG gene**. The 245 T/G (rs3134070) and 163 A/G (rs 3102735) SNPs appear to be in the same haplotype block (Promoter) but in different block (Exon 1) from the 1181 G/C (rs 2073618) SNP. The linkage between SNP pairs is shown in the figures as D'. The numbers in the figures represent the Levontin distance (100 × D'); numbers are omitted from SNP pairs showing a D' of 100. The correlation (r^2^) between the OPG loci studied was: rs3102735-rs2073618 = 0.01; rs3102735-rs3134070 = 0.39; rs3134070-rs2073618 = 0.09.

The SNPs to be analyzed, 163 A/G (rs3102735) located in promoter, 1181 G/C (rs2073618) located in exon I and 245 T/G (rs3134070) located in promoter of the *OPG *(chromosomal location 8q24, figure 1) [23] and the rs2277438 from the *RANKL *(13q14) located in a 5' untranslated region (UTR) [30] were chosen in relation to previous data in the literature suggesting possible associations with bone mass and functional significance. rs3102735 (163 A/G) is in strong linkage disequilibrium with other loci (rs3134070 (245 T/G), 950 T/C, and 6890 A/C) [22]. The *OPG *SNPs rs2073618 (1181 G/C) and rs3102735 (163 A/G) appear to be in different haplotype blocks (Figure 1). rs2073618 (1181 G/C) SNP of *OPG *causes the third amino acid of the signal peptide to change from lysine to asparagine. rs2277438 from the *RANKL *is a missense intronic substitution.

*Analysis of the OPG *rs3102735(*163 A/G)*, rs3134070 (*245 T/G) and *rs2073618 (*1181 G/C) polymorphisms*. Genotyping was performed with Custom Taqman^® ^SNP Genotyping assays (Assay-by-Design) (Applied Biosystems, Warrington, Cheshire, UK) using allele discrimination-specific Taqman probes, labelled with VIC and FAM. Oligonucleotide primers were designed, based on the sequence of the *OPG *available in GenBank (AB008821). The following primer sequences were used. For rs3102735 (163 A/G) SNP: CATGAATGGGACCACACTTTACAAG (forward) and TGCTCTAGGGTTCGCTGTCT (reverse). For rs3134070 (245 T/G) SNP: CCCTAGAGCAAAGTGCCAAACT (forward) and AGCTTCCTACGCGCTGAAC (reverse). For rs2073618 (1181 G/C SNP): CCAAGCCCCTGAGGTTTCC (forward) and CCCAGGGACTTACCACGAG (reverse).

*Analysis of RANKL rs2277438 polymorphism*. This SNP was assayed by validated Taqman SNP Genotyping Assay. TTGTTGGGGACATAAAGACTCTTGC[A/G]AGTATGAATTTTTTGTTCTTAAGTC (context sequence).

Linkage structure was established from the Hapmap Caucasian (CEU) population (http://www.Hapmap.org), analyzed with Haploview 4.2 software [31].

### Biochemical analysis

Fasting venous blood samples, for general biochemical analysis and specific determinations, were obtained after 30 minutes of supine rest from an antecubital vein. Samples were centrifuged immediately and serum was stored at -40°C in multiple aliquots until assayed to avoid freeze cycles. Total calcium (in serum and urine) and alkaline phosphatase were measured by automated methods in an ADVIA 2000 (Siemens Corp., Tarrytown, NY, USA). Intra- and inter-assay variations were <2%. Ionized calcium was measured by calcium-selective electrodes automated in a Ciba Corning 634 Ca^++^/pH Analyzer (Ciba Corning Diag. Corp, Medfield, Massachusetts, USA). Intact PTH was determined by automated immunoassay in a Liason (DiaSorin, Stillwater, Minnesota, USA). Sensitivity of assay was 5 pg/mL. Intra- and inter-assay variations were <5 and <8% respectively. 25-hydroxy-vitamin D was measured by RIA after extraction (DiaSorin, Stillwater, Minnesota, USA). The minimum detectable concentration was estimated to be 1.5 ng/mL. Intra- and inter-assay precision were 9.4 and 10.8%. Bone alkaline phosphatase (BAP) was determined by immunoassay (Alkphase B kit, Metra Biosystems, Mountain View, CA, USA). The minimum detectable dose is 0.7 U/L. Intra- and inter-assay variations were 3.5 and 6.2% respectively. Specificity: bone: 100%; liver: 3-8%; intestine: 0.4%. Serum osteocalcin was measured by immunoradiometric assay (IRMA) (OSTEO-RIACT kit, CIS Bio international, Gif-sur-Yvette, France). The sensitivity of the osteocalcin was 0.4 ng/mL. Intra- and inter-assay variations are 2.0 and 4.4% respectively. Collagen type I N-terminal propeptide levels were measured by RIA (Orion Diagnostica, Espoo, Finland). Sensitivity of the propeptide is 2 μg/L. Intra- and inter-assay variations are 8.75 and 5% respectively. Crosslaps were measured by ELISA (Nordic Bioscience Diagnostics, Herlev Hovedgade, Denmark). Sensitivity of assay is 0.010 ng/mL; intra- and inter-assay variations were 5.1 and 6.6% respectively. All biochemical and DXA procedures were performed at time of diagnosis and before any specific treatment had been initiated.

### Statistical analysis

The results were processed with the computer package SPSS (Statistical Package for Social Sciences, Chicago, IL, USA). Normal or non-normal distribution of the variables under study was tested with the Kolmogorov-Smirnov test. All data were expressed as mean ± SD (standard deviation of the mean), or the median and range for non-normal variables. Comparisons between PHPT patients and 114 age and gender matched controls from the 328 total control group were tested by Student *t*-test and Mann-Whitney U for normally and non-normally distributed parameters. Allele frequencies were estimated by counting, and the χ^2 ^test was used to identify significant departure from the Hardy-Weinberg equilibrium and for allele frequencies related to control and PHPT patients as well. In order to test association between the SNPs and PHPT, an odds ratio between cases and controls was calculated in addition. Fracture and nephrolithiasis frequency in the different genotype groups was tested by χ^2^. All shown data were adjusted for age, body mass index (BMI) and sex. Differences in age, BMI, BMD and bone remodelling markers between genotype groups were examined using the One-way ANOVA or Kruskal-Wallis test. If global test results were significant, between-group differences were then tested after Bonferroni correction for multiple comparisons (with a cut-off p value of 0.0167) except for BMD 1/3 radius and osteocalcin. In these two cases we used the Mann-Whitney U test (with a confidence interval of 98.3%) between pairs of alleles because there was only one subject in GG group who had osteocalcin and BMD 1/3 radius studied.

## Results

General characteristics, bone biochemical parameters (non normal distribution) and BMD measurements (normal distribution) of both patients and control subjects are shown in Table 1. The presence of PHPT was excluded in all of the control subjects by measuring calcium and PTH. There were no differences regarding age, sex and menopause status between groups. The 25-OH-Vit D status was similar in both groups. BMI was significantly higher in PHPT than in control group. As expected, we found significant high levels of bone biochemical parameters in the PHPT group compared with the control group and lower BMD in the PHPT patients than in the control subjects at the three studied sites.

**Table 1:** General features of control and PHPT subjects

	Control group	PHPT group	p
	(n = 114^+^)	(n = 298)	
**Age **(years)	59 ± 13	62 ±13	0.052

♀/♂	5/1	5.6/1	0.661

**Menopause **♀ (%)	87.2	89.5	0.536

**BMI **(kg/m^2^)	25.3 ± 3.8	28.7 ± 5.0	**0.000**

**Fracture **(%)	21	24.6	**0.000**

**Renal lithiasis **(%)	6	36.0	**0.000**

**25-OH-VitD **(ng/mL )	20.4 (6-42)	17.2 ± (3-93)	0.997

**25-OH-VitD **≥ **30 ng/mL **(%)	15.2	9.2	0.221

**Creatinine **(mg/dL)	0.98 ± 0.14	1,02 ± 0.29	0.179

**Total Calcium **(mg/dL)	9.4 (8.6-10.6)	10.7 (9.8-14.5)	**0.000**

**Ionized Calcium **(mM)	1.23 (1.11-1.36)	1.47 (1.25-2.07)	**0.000**

**PTH **(pg/mL)	37 (10-105)	179 (59-1279)	**0.000**

**Alk Phosphatase **(U/L)	63.4 (33-198)	82.3 (20-373)	**0.000**

**B Alk Phosphatase **(U/L)	22.1 (11-53)	32.9 (5-85)	**0.000**

**P1NP **(μg/L)	46.3 (15-145)	54.9 (11-255)	**0.005**

**Crosslaps B **(ng/mL)	0.475 (0.053-1.460)	0.814 (0.044-3.208)	**0.000**

**Osteocalcin **(ng/mL)	19.4 (6-39)	30.9 (7-175)	**0.000**

**Serum OPG **(pmol/L)	3.0 (0.89-5.61)	4.8 (0.01-12.42)	**0.000**

**LS_BMD **(g/cm^2^)	0.935 ± 0.134	0.879 ± 0.155	**0.000**

**FemNeck_BMD **(g/cm^2^)	0.749 ± 0.120	0.691 ± 0.104	**0.000**

**TotHip_BMD **(g/cm^2^)	0.887 ± 0.126	0.840 ± 0.145	**0.000**

**Rad 1/3_BMD **(g/cm^2^)	0.671 ± 0.696	0.629 ± 0.102	**0.000**

Data shown as mean ± SD or median and range for non normal variables. B Alk: bone alkaline. OPG: osteoprotegerin. LS: lumbar spine. Fem. Neck: femoral neck. Tot Hip: total hip. 1/3 Rad: third distal radius. BMD: bone mineral density. T Score: BMD related to peak bone mass. Z score: BMD related to mean BMD in general population of equal age and gender. ^**+**^The 114 control subjects were a subgroup from the 328 total control group age and gender matched with the HPP patients.

Allelic frequencies did not deviate from Hardy-Weinberg equilibrium. Genotype frequencies for controls and primary hyperparathyroidism subjects showed no differences in any of the SNPs studied (Tables 2 and 3).

**Table 2:** Allelic frequencies in both control and PHPT subjects

	*OPG *rs3102735 163 A/G			*OPG *rs3134070 245 T/G			*OPG*rs2073618 1181 G/C			*RANKL*_rs2277438		
	
	AA	AG	GG	TT	TG	GG	GG	GC	CC	AA	AG	GG
**Control = 328**	73.8%	24.3%	1.9%	91.6%	7.6%	0.4%	26.2%	47.1%	26.8%	67.5%	30.8%	1.7%

**PHPT = 298**	70.2%	27.6%	2.2%	90.1%	9.3%	0.6%	27.1%	44.5%	28.3%	74.1%	24.3%	1.6%

Chi^2 ^: not statistically significant in any of the SNPs.

**Table 3:** Odds ratio between control and PHPT subjects

	*OPG *rs3102735		*OPG *rs3134070		*OPG *rs2073618		*RANKL*_rs2277438	
	
	A	G	T	G	G	C	A	G
**Control = 328**	564	92	625	29	326	330	543	113

**PHPT = 298**	500	96	564	32	295	301	514	82
	OR = 1.18 CI [0.86-1.6]		OR = 1.22 CI [0.82-1.79]		OR = 1.01 CI [0.73-1.26]		OR = 1.06 [0.77-1.44]	

OR: Odds Ratio. CI : confidence interval.

Regarding the age of diagnosis, BMI, menopause status, frequency of fractures or renal lithiasis, we found no differences between genotypes in any of the SNPs studied in the PHPT group (Additional File 1) nor in control subjects.

### Relationship between the different SNPs studied and BMD and bone remodelling markers

#### SNP 163 A/G *OPG *(rs 3102735)

In PHPT patients, BMD at the 1/3 distal radius was significantly lower in the minor allele homozygote group (GG) versus the other two groups (p = 0.039); (GG-AA p = 0.038; GG-AG p = 0.037) (Table 4). There were no differences between the major allele homozygote and heterozygote group. Analysis of BMD in the lumbar spine or hip did not show any difference between genotypes. PTH, total and ionized calcium, P1NP, β Crosslaps, osteocalcin and bone alkaline phosphatase (BAP) showed a trend (not statistically significant) to higher levels in the minor allele homozygote group versus the other two groups. There were no differences in vitamin D levels among the three groups (data not shown except for PTH (Table 4, Additional File 2). These results were not different in the subgroup of women and we found no difference in their menopausal status between the three genotype groups.

**Table 4:** Comparison between genotypes of the SNP 163 A/G OPG rs3102735 and 245 T/G OPG rs3134070 in PHPT patients

	163 A/G *OPG *rs3102735		p	245 T/G *OPG *rs3134070		p
	*AA*	61.7 ± 12.4		*TT*	61.2 ± 12.8	
				
**Age**	*AG*	61.0 ± 14.2	0.449	*TG*	64.3 ± 14.7	0.298
				
(years)	*GG*	67.8 ± 15.2		*GG*	70.5 ± 7.7	

	*AA*	28.8 ± 5.1		*TT*	28.6 ± 4.9	
				
**BMI**	*AG*	28.4 ± 4.7	0.793	*TG*	29.5 ± 5.1	0.663
				
(kg/m^2^)	*GG*	28.6 ± 3.5		*GG*	28.6 ± 3.5	

	*AA*	1.02 ± 0.32		*TT*	1.01 ± 0.30	
				
**Creatinine**	*AG*	1.02 ± 0. 22	0.580	*TG*	1.05 ± 0.23	0.836
				
(mg/dL)	*GG*	0.89 ± 0.16		*GG*	1.03 ± 0.05	

	*AA*	140 (32-873)		*TT*	134 (32-873)	
				
**PTH**	*AG*	144 (50-1279)	0.927	*TG*	203 (55-1279)	0.314
				
(pg/mL)	*GG*	164 (61-747)		*GG*	252 (132-373)	

	*AA*	0.879 ± 0.142		*TT*	0.869 ± 0.142	
				
**LS BMD**	*AG*	0.863 ± 0.147	0.473	*TG*	0.919 ± 0.162	0.234
				
(g/cm^2^)	*GG*	0.826 ± 0.267		*GG*	0.672	

	*AA*	0.680 ± 0.104		*TT*	0.680 ± 0.102	
				
**Fem. Neck**	*AG*	0.685 ± 0.092	0.136	*TG*	0.710 ± 0.090	0.151
				
**BMD **(g/cm^2^)	*GG*	0.751 ± 0.139		*GG*	0.747 ± 0.053	

	*AA*	0.834 ± 0.144		*TT*	0.833 ± 0.140	
				
**Total Hip**	*AG*	0.831 ± 0.128	0.241	*TG*	0.848 ± 0.134	0.484
				
**BMD **(g/cm^2^)	*GG*	0.906 ± 0.135		*GG*	0.881 ± 0.008	

	*AA*	0.629 ± 0.096	**<0.05**	*TT*	0.616 ± 0.097	**<0.05**
				
**1/3 Rad**	*AG*	0.635 ± 0.103	**AA-GG: 0,038**	*TG*	0.654 ± 0.100	**GG-TT: 0,003**
				
**BMD **(g/cm^2^)	*GG*	0.501 ± 0.147	**AG-GG: 0,037**	*GG*	0.326	**GG-TG: 0,01**

Data shown as mean ± SD or median and range for non normal variables. LS: lumbar spine. Fem. Neck: femoral neck. 1/3 Rad: third distal radius. BMD: bone mineral density. Number of subjects: OPG 163: AA = 205, AG = 86, GG = 6. OPG 245

TT = 266, TG = 30, GG = 2 (except for LS BMD and 1/3 Rad BMD GG data = 1 subject).

In the control group, we did not find any difference between genotypes regarding BMD at three sites (Additional File 3).

#### SNP 245 T/G *OPG *(rs3134070)

In PHPT patients, BMD in the 1/3 distal radius was significantly lower in the minor allele (GG) homozygote group versus the other two groups despite its low frequency (p = 0.003); (GG-TT p = 0.003 and GG-TG p = 0.01) (Table 4). Analysis of BMD in the lumbar spine or hip did not show any difference between genotypes. Total calcium (mg/dL) GG = 12.3 ± 0.9; TT = 10.7 ± 0.7; TG = 10.9 ± 1.1 (p = 0.011); GG-TT p = 0.012 and GG-TG p = 0.032, β Crosslaps (ng/mL) GG = 1.770 ± 2.033; TT = 0.816 ± 0.512; TG = 0.726 ± 0.356 (p = 0.033); GG-TT p = 0.035 and GG-TG p = 0.029 and osteocalcin (ng/mL) GG = 175.0; TT = 30.4 ± 19.0; TG = 30.6 ± 18.2 (p < 0.001); GG-TT p < 0.001 and GG-TG p < 0.001 levels were significantly higher in the minor homozygote allele group than in the other two groups (Additional File 2). These results were not different in the subgroup of women and we found no difference in their menopausal status between the three genotype groups.

In the control group, we did not find any difference between genotypes regarding BMD in the three sites (Additional File 3).

#### SNP 1181 G/C *OPG *(rs 2073618)

In PHPT patients, BMD at all sites and bone remodelling markers were similar in the three genotype groups (Additional File 2). These results were not different in the subgroup of women and we found no difference in their menopausal status between the three genotype groups.

In the control group, we found higher levels of BMD (g/cm^2^) in CC subjects *vs *in GG (0.983 ± 0.170 *vs *0.872 ± 0.155; p = 0.028) in the lumbar spine (Additional File 3).

#### SNP *RANKL *rs2277438

In PHPT patients, we found a non significant trend towards lower BMD in the 1/3 distal radius (g/cm^2^) (AA = 0.622 ± 0.105; AG = 0.610 ± 0.087; GG = 0.543 ± 0.074; p = 0.835), to lower BMD at total hip (g/cm^2^) (AA = 0.841 ± 0.132; AG = 0.821 ± 0.160; GG = 0.791 ± 0.118; p = 0.502) and to higher levels of BAP (U/L) (AA = 33.5 ± 17.3; AG = 29.9 ± 13.0; GG = 43.7 ± 8.4; p = 0.122) in the minor allele homozygote group (GG) versus the other two groups (Additional File 2). These results were not different in the subgroup of women and we found no difference in their menopausal status between the three genotype groups.

In the control group, we did not find any differences between genotypes regarding BMD at three sites (Additional File 3).

Due to the low frequency of one of the homozygous genotypes in the *OPG *rs3102735 (163 A/G), *OPG *rs3134070 (245 T/G) and *RANKL *rs2277438, we grouped the heterozygote and the less frequent homozygous subjects. We did not find any differences between the groups regarding BMD in all sites.

## Discussion

As expected, subjects in the PHPT group had high levels of calcium, PTH, bone remodelling markers and low BMD at the three studied sites. We found BMI corresponding to overweight in the PHPT group consistent with previous studies [32,33] although the nature of this relationship remains uncertain. We found no differences in vitamin D levels between patients and control subjects, as Vignali had also found in postmenopausal PHPT women [34], and, in contrast with previous data [35] showing lower vitamin D levels in PHPT patients than in control subjects, we did not find such a difference between the study groups although we did not evaluate seasonal factors as the aforementioned study did. OPG serum levels were three times higher in our PHPT patients than in a previous study on men and women aged 62 with PHPT [36] and our PHPT subjects had lower levels than a group of postmenopausal women [37]. Despite there being no established reference values, OPG serum levels appear to be higher in postmenopausal women than in other conditions as PHPT.

There were no differences in the allelic distribution between PHPT and control groups, this means that none of the studied SNPs appears to be a genetic factor which predisposes for PHPT.

The main purpose of this study was to analyze the relationship between BMD, or fractures, and three SNPs of the *OPG *and the rs2277438 SNP of the *RANKL *in PHPT. We did not find any differences in frequency of fractures between genotypes in control nor in PHPT subjects in all the SNPs studied according to data of previous studies of *OPG *rs3102735 (163 A/G) [27] and of *OPG *rs2073618 (1181 G/C) [27,28] on postmenopausal women. These two studies neither did find any differences in BMD. A study of *OPG *rs2073618 (1181 G/C) described a 26% higher risk of hip fractures and 52% higher of femoral neck fractures in CC homozygote than GG homozygote women independent of BMD [24]. There are no fracture data related to *RANKL *rs2277438 SNP in the literature. The genetic variation that causes SNPs appear not to have main influence on a clinical event as relevant as fractures in PHPT and in non PHPT population.

Significantly lower BMD in the 1/3 distal radius with similar PTH levels were found in the minor homozygotes (GG) compared to heterozygotes and major allele homozygotes in both *OPG *rs3102735 (163 A/G) and *OPG *rs3134070 (245 T/G) SNPs in PHPT but not in control subjects. This could mean that these minor homozygote individuals suffer from more specific cortical BMD loss not mediated by PTH or creatinine serum levels in PHPT. An association between the G allele of the OPG rs3102735 (163 A/G) SNP and low BMD in the forearm, low heel broadband ultrasound attenuation (BUA) and low heel speed of sound (SOS) were described in Danish women with and without hip or forearm fractures [16]. In Hungarian women the GG genotype of the *OPG *rs3102735 (163 A/G) was associated with low hip BMD [17]. Nevertheless, Hsu reported that males with the GG genotype of the *OPG *rs3102735 (163 A/G) had very low risk of having extremely low BMD in the hip [30]. Regarding the *OPG *rs3134070 (245 T/G) SNP, an association between the GG genotype and low BMD has already been reported in the radius and femoral neck in postmenopausal women [18]. Several other studies failed to show this association between *OPG *rs3102735 (163 A/G) or *OPG *245 T/G [19,22,27,28,38].

We did not find any difference between genotypes of the *OPG *rs2073618 (1181 G/C) SNP regarding BMD in the PHPT subjects, a finding in accordance with previous studies on menopausal women [27,28]. However, we did find higher BMD in the lumbar spine in the CC than in the GG genotype group in the healthy control subjects, also in accordance with previous findings in the general or postmenopausal osteoporotic populations [15,19,21-24]. These findings could mean that the bone loss in PHPT, mainly cortical, and in the osteoporotic condition, mainly cancellous, is modulated by different genetic factors. Some possible factors could be the G allele of the *OPG *rs2073618 (1181 G/C) SNP favouring lumbar spine bone loss in osteoporosis, or the G allele of the *OPG *rs3134070 (245 T/G) and rs3102735 (163 A/G) favouring cortical bone loss in PHPT. But it should be also considered that the wrist is a non-weight bearing site and therefore is free of external factors or remodelling due to body weight or physical activity.

Consistent with studies in the non-PHPT population [19,29,30] we did not find any significant difference between genotypes of the *RANKL *rs2277438 SNP in PHPT or in the control group.

The main limitations of our study are the low frequency of the GG genotype in both the *OPG *rs3102735 (163 A/G) and rs3134070 (245 T/G) SNPs and that there was a trend (not statistically significant) to difference in age between the GG and the rest of the groups. Otherwise, our results are supported by the fact that they are not reproducible in control subjects and that these two SNPs are located in the same haplotype block (promoter).

## Conclusion

Our study provides the first evaluation of the relationship between SNPs of the OPG/RANK system and sporadic PHPT. Subjects with PHPT and GG genotype for the *OPG *rs3102735 (163 A/G) and *OPG *rs3134070 (245 T/G) SNPs appear to be at risk of having a lower BMD in the distal radius, which is mainly cortical bone. This association did not appear to be mediated by any difference in PTH serum levels. Further studies on larger PHPT populations, with complementary functional evaluation, are needed to support our results.

## List of the abbreviations

P1NP: Amino-terminal propeptyde type 1 Colagen; BMI: Body mass index BAP: Bone alkaline phosphatase; BMD: Bone mineral density; IRMA: Immunoradiometric assay; RANKL: Ligand of the receptor activator of nuclear factor-κβ OP: Osteoporosis; OPG: Osteoprotegerin; PTH: Parathyroid hormone; PCR: Polymerase chain reaction; PHPT: Primary hyperparathyroidism; RANK: Receptor activator of nuclear factor-κβ; SNP: Single nucleotide polymorphism; ELISA: Specific immunoassay; SD: Standard deviation of the mean; SPSS: Statistical Package for Social Sciences; DXA: X-ray absorptiometry.

## Competing interests

The authors declare that they have no competing interests.

## Authors' contributions

all authors have read and approved the final manuscript.

MP: made substantial contributions to conception, design and draft of the manuscript and in acquisition,

analysis and interpretation of data

MTG-U: was involved in drafting, conception and design of the manuscript.

AB: carried out molecular genetic studies

BP: carried out inmunoassays

BAL: carried out inmunoassays

CV: carried out the double X ray absorptiometries

JAR: revised the manuscript critically for important intellectual content

JAA: was involved in drafting the manuscript and gave final approval of the version to be published

## Pre-publication history

The pre-publication history for this paper can be accessed here:

http://www.biomedcentral.com/1471-2350/12/168/prepub

## Supplementary Material

Additional file 1 **"Distribution Of Fractures And Lithiasis Frequency Among the Genotypes of the Snps Studied In Phpt Patients"**. This file contains two tables showing the distribution of fractures and lithiasis frequency among the genotype groups of the *OPG *163 A/G rs3102735, *OPG *245 T/G rs3134070, *OPG *1181 G/C rs2073618 and *RANKL *rs2277438 in PHPT patients. Click here for file

Additional file 2 **"Comparison Of Biochemical Parameters Among The Genotype Groups of the Snps Studied In Phpt Subjects"**. This file contains several tables showing the comparison of biochemical parameters among the genotype groups of the *OPG *163 A/G rs3102735, *OPG *245 T/G rs3134070, *OPG *1181 G/C rs2073618 and *RANKL *rs2277438 in PHPT patients. Click here for file

Additional file 3 **"Bone Mineral Density Distribution Among The Genotypes of the Snps Studied In Control Subjects"**. This file contains a table showing the distribution of BMD levels among the three genotype groups of the *OPG *163 A/G rs3102735, *OPG *245 T/G rs3134070, *OPG *1181 G/C rs2073618 and *RANKL *rs2277438 in the control subjects. Click here for file
