# Supplementary material for: "Single nucleotide polymorphisms of the OPG/RANKL system genes in primary hyperparathyroidism and their relationship with bone mineral density"
Source: BMC Med Genet. 2011 Dec 20;12:168. doi: 10.1186/1471-2350-12-168 (PMC3267665; doi:10.1186/1471-2350-12-168)
Supplement: Additional file 1 — "Distribution Of Fractures And Lithiasis Frequency Among the Genotypes of the Snps Studied In Phpt Patients". This file contains two tables showing the distribution of fractures and lithiasis frequency among the genotype groups of the OPG 163 A/G rs3102735, OPG 245 T/G rs3134070, OPG 1181 G/C rs2073618 and RANKL rs2277438 in PHPT patients. [file 1471-2350-12-168-S1.DOC]

**Distribution of fractures and lithiasis frequency among the genotypes of the SNPs studied in PHPT patients**

**Distribution of lithiasis frequency among the genotypes of the SNPs studied in PHPT patients**

|  |  | Lithiasis | No Lithiasis |
| --- | --- | --- | --- |
| *OPG* 163 A/G rs3102735 | **AA** | 34,5 % | 65,5 % |
|  | **AG** | 40 % | 60 % |
|  | **GG** | 28,6 % | 71,4 % |
| *OPG* 245 T/G rs3134070 | **TT** | 35,2 % | 64,8 % |
|  | **TG** | 40,7 % | 59,3 % |
|  | **GG** | 100 % | 0 |
| *OPG* 1181 G/C rs2073618 | **GG** | 38,7 % | 61,3 % |
|  | **GC** | 35,2 % | 64,8 % |
|  | **CC** | 34,9 % | 65,1 % |
| *RANKL* rs2277438 | **AA** | 37,6 % | 62,4 % |
|  | **AG** | 31,9 % | 68,1 % |
|  | **GG** | 33,3 % | 66,7 % |

All comparisons were not statistically significant

**Distribution of fractures frequency among the genotypes of the SNPs studied in PHPT patients**

|  | | Fractures | No Fractures |
| --- | --- | --- | --- |
| *OPG* 163 A/G rs3102735 | **AA** | 25,4 % | 74,6 % |
|  | **AG** | 25,3 % | 74,7 % |
|  | **GG** | 0 | 100 % |
| *OPG* 245 T/G rs3134070 | **TT** | 25,4 % | 74, 6 % |
|  | **TG** | 20 % | 80 % |
|  | **GG** | 0 | 100 % |
| *OPG* 1181 G/C rs2073618 | **GG** | 20,3 % | 79,7 % |
|  | **GC** | 29,2 % | 70, 8 % |
|  | **CC** | 22,4 % | 77,6 % |
| *RANKL* rs2277438 | **AA** | 25,1 % | 74,9 % |
|  | **AG** | 24 % | 76 % |
|  | **GG** | 25 % | 75 % |

All comparisons were not statistically significant
