# Supplementary material for: "Single nucleotide polymorphisms of the OPG/RANKL system genes in primary hyperparathyroidism and their relationship with bone mineral density"
Source: BMC Med Genet. 2011 Dec 20;12:168. doi: 10.1186/1471-2350-12-168 (PMC3267665; doi:10.1186/1471-2350-12-168)
Supplement: Additional file 2 — "Comparison Of Biochemical Parameters Among The Genotype Groups of the Snps Studied In Phpt Subjects". This file contains several tables showing the comparison of biochemical parameters among the genotype groups of the OPG 163 A/G rs3102735, OPG 245 T/G rs3134070, OPG 1181 G/C rs2073618 and RANKL rs2277438 in PHPT patients. [file 1471-2350-12-168-S2.DOC]

**COMPARISON OF BIOCHEMICAL PARAMETERS AMONG THE GENOTYPE GROUPS OF THE SNP STUDIED IN PHPT SUBJECTS**

**Comparison of biochemical parameters among the three genotypes of *OPG* 163 A/G rs3102735 in PHPT patients**

| *OPG* 163 A/G rs3102735 | | Median and range | p |
| --- | --- | --- | --- |
| Total calcium **(mg/dL)** | **AA** | 10.7 (8.7-13.5) | **0.041 (+)** |
|  | **AG** | 11.0 (8.5-14.5) |  |
|  | **GG** | 10.5 (9-13) |  |
| Ionized calcium **(mM)** | **AA** | 1.50 (1.21-2.7) | 0.198 |
|  | **AG** | 1.50 (1.20-1.96) |  |
|  | **GG** | 1.40 (1.31-1.51) |  |
| 25-OH-Vitamin D **(ng/mL)** | **AA** | 17 (3-93) | 0.252 |
|  | **AG** | 18 (4-47) |  |
|  | **GG** | 15 (8-24) |  |
| Alkaline Phosphatase **(U/L)** | **AA** | 80 (20-373) | 0.723 |
|  | **AG** | 87 (45-302) |  |
|  | **GG** | 88 (46-191) |  |
| Serum OPG **(pmol/L)** | **AA** | 4.8 (0.01-12.4) | 0.735 |
|  | **AG** | 4.9 (2.01-10.00) |  |
|  | **GG** | 3.9 (2.39-5.6) |  |
| P1NP **(µg/L)** | **AA** | 55 (11-255) | 0.826 |
|  | **AG** | 53 (16-140) |  |
|  | **GG** | 63 (17-155) |  |
| β Crosslaps **(ng/mL)** | **AA** | 0.829 (0.044-2.853) | 0.506 |
|  | **AG** | 0.762 (0.192-2.182) |  |
|  | **GG** | 1.041 (0.333-3.208) |  |
| Bone Alkaline Phosphatase **(U/L)** | **AA** | 32 (5-79) | 0.081 |
|  | **AG** | 33 (12-84) |  |
|  | **GG** | 49 (19-85) |  |
| Osteocalcin **(ng/mL)** | **AA** | 31 (7-126) | **<0.001**  ***AG-GG <0.001***  ***AA-GG <0.001*** |
|  | **AG** | 29 (7-67) |  |
|  | **GG** | 79 (19-175) |  |

**Comparison of biochemical parameters among the three genotypes of *OPG* 245 T/G rs3134070 in PHPT patients**

| *OPG* 245 T/G rs3134070 | | Median and range | p |
| --- | --- | --- | --- |
| Total calcium **(mg/dL)** | **TT** | 10.7 (8.7-13.5) | **0.011**  ***GG-TT= 0.012***  ***GG-TG= 0.032*** |
|  | **TG** | 10.9 (8.5-14.5) |  |
|  | **GG** | 12.9 (11.7-13) |  |
| 25-OH-Vitamin D **(ng/mL)** | **TT** | 17.0 (3-93) | 0.702 |
|  | **TG** | 17.5 (6.6-39.7) |  |
|  | **GG** | 14.5 (12.0-17.0) |  |
| Ionized calcium **(mM)** | **TT** | 1.47 (1.21-2.07) | 0.565 |
|  | **TG** | 1.51 (1.20-1.96) |  |
|  | **GG** | 1.51 |  |
| Alkaline Phosphatase **(U/L)** | **TT** | 81 (20-373) | 0.301 |
|  | **TG** | 89 (55-156) |  |
|  | **GG** | 122,5 (54-191) |  |
| Serum OPG **(pmol/L)** | **TT** | 4.8 (0.01-12.42) | 0.957 |
|  | **TG** | 5.1 (3.72-9.2) |  |
|  | **GG** | 4.8 (4.00-5.6) |  |
| P1NP **(µg/L)** | **TT** | 54 (11-255) | 0.204 |
|  | **TG** | 62 (18-110) |  |
|  | **GG** | 91 (27-155) |  |
| β Crosslaps **(ng/mL)** | **TT** | 0.816 (0.44-2.853) | **0.033**  ***GG-TT= 0.035***  ***GG-TG = 0.029*** |
|  | **TG** | 0.726 (0.192-1.342) |  |
|  | **GG** | 1.77 (0.333-3.208) |  |
| Bone Alkaline Phosphatase **(U/L)** | **TT** | 33 (5-84) | 0.196 |
|  | **TG** | 35 (12-63) |  |
|  | **GG** | 52 (19-85) |  |
| Osteocalcin **(ng/mL)** | **TT** | 30.5 (7-126) | **<0.001**  ***GG-TT<0.001***  ***GG-TG<0.001*** |
|  | **TG** | 30.6 (7.2-67) |  |
|  | **GG** | 175 |  |

**Comparison of biochemical parameters among the three genotypes of *OPG* 1181 G/C rs2073618 in PHPT patients**

| *OPG* 1181 G/C rs2073618 | | | Median and range | | p |  |
| --- | --- | --- | --- | --- | --- | --- |
| Total calcium **(mg/dL)** | **GG** | 10.9 (9.1-14.5) | | 0.438 | | |
|  | **GC** | 10.7 (8.5-13) | |  |  |  |
|  | **CC** | 10.8 (9-13) | |  |  |  |
| 25-OH-Vitamin D **(ng/mL)** | **GG** | 17 (3-50) | | 0.892 | | |
|  | **GC** | 17 (4-93) | |  |  |  |
|  | **CC** | 17 (3-55) | |  |  |  |
| Ionized calcium **(mM)** | **GG** | 1.47 (1.20-1.96) | | 0.254 | | |
|  | **GC** | 1.47 (1,25-1.79) | |  |  |  |
|  | **CC** | 1.48 (1.21-2.07) | |  |  |  |
| Alkaline Phosphatase **(U/L)** | **GG** | 83 (21-191) | | 0.162 | | |
|  | **GC** | 87 (20-373) | |  |  |  |
|  | **CC** | 73 (29-302) | |  |  |  |
| Serum OPG **(pmol/L)** | **GG** | 5.0 (0.01-11.53) | | 0.566 | | |
|  | **GC** | 4.7 (1.21-10) | |  |  |  |
|  | **CC** | 4.7 (2.1-12.42) | |  |  |  |
| P1NP **(µg/L)** | **GG** | 63 (21-155) | | 0.518 | | |
|  | **GC** | 52 (18-255) | |  |  |  |
|  | **CC** | 51 (11-142) | |  |  |  |
| β Crosslaps **(ng/mL)** | **GG** | 0.977 (0.210-3.208) | | **0.044** | | |
|  | **GC** | 0.777 (0.192-2.500) | |  |  |  |
|  | **CC** | 0.747 (0.044-3.208) | |  |  |  |
| Bone Alkaline Phosphatase **(U/L)** | **GG** | 39 (12-85) | | **0.016**  ***GG-GC=0.01***  ***GG-CC=0.018*** | | |
|  | **GC** | 31 (5-84) | |  |  |  |
|  | **CC** | 31 (12-72) | |  |  |  |
| Osteocalcin **(ng/mL)** | **GG** | 39 (9-175) | | **0.009**  ***GG-GC=0.004*** | | |
|  | **GC** | 27 (7-62) | |  |  |  |
|  | **CC** | 30 (7-80) | |  |  |  |

**Comparison of biochemical parameters among the three genotypes of *RANKL* rs2277438 in PHPT patients**

| *RANKL* rs2277438 | | Median and range | p |
| --- | --- | --- | --- |
| Total calcium **(mg/dL)** | **AA** | 10.8 (9-14.5) | 0.339 |
|  | **AG** | 10.6 (8.5-11.8) |  |
|  | **GG** | 10.5 (10-10.9) |  |
| 25-OH-Vitamin D **(ng/mL)** | **AA** | 17 (3-93) | 0.594 |
|  | **AG** | 17 (4-55) |  |
|  | **GG** | 15 (12-21) |  |
| Ionized calcium **(mM)** | **AA** | 1.47 (1.20-2.07) | 0.351 |
|  | **AG** | 1.44 (1.20-1.74) |  |
|  | **GG** | 1.48 (1.37-1.57) |  |
| Alkaline Phosphatase **(U/L)** | **AA** | 82 (20-373) | 0.956 |
|  | **AG** | 82 (21-339) |  |
|  | **GG** | 76 (45-111) |  |
| Serum OPG **(pmol/L)** | **AA** | 4.85 (0.01-11.44) | 0.411 |
|  | **AG** | 4.77 (1.21-12.42) |  |
|  | **GG** | 5.63 (4.3-7.5) |  |
| P1NP **(µg/L)** | **AA** | 53 (11-192) | 0.954 |
|  | **AG** | 55 (10-255) |  |
|  | **GG** | 52 (39-68) |  |
| β Crosslaps **(ng/mL)** | **AA** | 0.808 (0.117-3.208) | 0.847 |
|  | **AG** | 0.776 (0.044-2.853) |  |
|  | **GG** | 0.867 (0.600-1.228) |  |
| Bone Alkaline Phosphatase **(U/L)** | **AA** | 33 (5-86) | 0.122 |
|  | **AG** | 31 (12-80) |  |
|  | **GG** | 44 (36-55) |  |
| Osteocalcin **(ng/mL)** | **AA** | 30 (7-175) | 0.975 |
|  | **AG** | 30 (6-126) |  |
|  | **GG** | 26 (14-44) |  |
