# Supplementary material for: "Single nucleotide polymorphisms of the OPG/RANKL system genes in primary hyperparathyroidism and their relationship with bone mineral density"
Source: BMC Med Genet. 2011 Dec 20;12:168. doi: 10.1186/1471-2350-12-168 (PMC3267665; doi:10.1186/1471-2350-12-168)
Supplement: Additional file 3 — "Bone Mineral Density Distribution Among The Genotypes of the Snps Studied In Control Subjects". This file contains a table showing the distribution of BMD levels among the three genotype groups of the OPG 163 A/G rs3102735, OPG 245 T/G rs3134070, OPG 1181 G/C rs2073618 and RANKL rs2277438 in the control subjects. [file 1471-2350-12-168-S3.DOCX]

**Bone mineral density distribution among the genotypes of the SNPs studied in control subjects**

|  | | BMD LS  (g/cm^2^) | BMD Fem Neck  (g/cm^2^) | BMD Total hip  (g/cm^2^) | BMD 1/3 Rad (g/cm^2^) |
| --- | --- | --- | --- | --- | --- |
| *OPG*  163 A/G | **AA** | 0.980 ± 0.137 | 0.805 ± 0.134 | 0.942 ± 0.147 | 0.693 ± 0.076 |
|  | **AG** | 0.997 ± 0.127 | 0.820 ± 0.132 | 0.948 ± 0.126 | 0.697 ± 0.089 |
|  | **GG** | 1.049 ± 0.155 | 0.872 ± 0.098 | 0.981 ± 0.083 | 0.734 ± 0.106 |
| *OPG*  245 T/G | **TT** | 0.974 ± 0.136 | 0.777 ± 0.119 | 0.908 ± 0.129 | 0.696 ± 0.077 |
|  | **TG** | 0.936 ± 0.130 | 0.760 ± 0.131 | 0.873 ± 0.095 | 0.685 ± 0.109 |
|  | **GG** | 0.994 | 0.763 | 0.870 | 0.662 |
| *OPG*  1181 G/C | **GG** | 0.872 ± 0.155 **(*+)*** | 0.802 ± 0.135 | 0.937 ± 0.141 | 0.694 ± 0.075 |
|  | **GC** | 0.977 ± 0.129 **(*+)*** | 0.813 ± 0.133 | 0.947 ± 0.142 | 0.693 ± 0.083 |
|  | **CC** | 0.983 ± 0.170 **(*+)*** | 0.811 ± 0.130 | 0.950 ± 0.140 | 0.670 ± 0.077 |
| *RANKL*  rs2277438 | **AA** | 0.974 ± 0.129 | 0.781 ± 0.123 | 0.905 ± 0.127 | 0.696 ± 0.082 |
|  | **AG** | 0.962 ± 0.150 | 0.766 ± 0.115 | 0.901 ± 0.125 | 0.692 ± 0.073 |
|  | **GG** | 1.010 | 0.818 | 1.082 | **n=0** |

***(+)****Anova p=0,028 Bonferroni correction for GG-CC p=0,033.* ***(*)*** *Anova p=0,065. All the other comparisons were not statistically significant. Data expressed as mean ± SD. LS: lumbar spine. Fem neck: femoral neck. Rad 1/3: distal radius. BMD: bone mineral density.*
